# A Letter from George Pearson, M. D. Concerning a Report of Two Persons Having Died under the Cow-Pock Inoculation

**Published:** 1800-05

**Authors:** G. Pearson

**Affiliations:** Liecester Square


### *A Letter from* George Pearson, M. D. *concerning a Report of two Persons having died under the Cow-Pock Inoculation.*

A REPORT continuing to be propagated in fome circles of high rank, that two perfons had died at Petworth, under *the inoculated Cow-pock*, with matter which I fent from London, I think it my duty to undeceive the public, in order that the credit of the new practice may not, by this means, fuffer undefervedly.

In the inoculation alluded to, at Petworth, I found that the matter matter employed had been taken from some inoculated patients at Brighthelmstone, who had an eruption resembling the Small-pox; and that the variolous-like disease in them had been excited by matter taken from certain patients in Brighthelmstone, who also had variolous-like eruptions, and who had been inoculated with matter taken, not by myself but by a surgeon of unquestionable accuracy from one of my patients in the distinctly marked Cow-pock, without such eruptions.

Being desirous of avoiding controversy, and being even unwilling, at present, to enter into a theoretical discussion of the nature of these eruptive cases, I apprehend it is only necessary to avail myself of the liberty given to me of publishing the Letter of the respectable surgeon who had the care of the patients at Petworth, and which was written before it was supposed that it would be proper to lay it before the public.

Letter of Mr. ANDRE to Dr. PEARSON.

"SIR,

"THE matter sent from Brighton to Petworth produced a disease in every shape resembling the Small-pox: the time of sickening, the symptoms, the eruptions and their maturation were the same. The number inoculated was *fourteen*. Three of these were children at the breast; the number of eruptions in them was from three to twelve. The ages of the remaining eleven were from three to fourteen, and the numbers of the eruptions from fifty to a thousand. I shall be very glad to give you any further information in my power, should you have any more questions to propose.

"I remain, your obedient humble servant,

*Petworth, Dec.* 11, 1799. "W. ANDRE."

I think it necessary to state, in addition to the above letter of Mr. Andre, that I have lately learned, by a letter which Lord Egremont, as well as Mr. Andre, did me the honour to write, that during the inoculation at Petworth, "A woman caught the Small-pox *in the natural way*, and died; but it could not be clearly traced, whether she was infected from the inoculated patients: She might, or she might not."—Mr. Andre's Letter, April 13, 1800.

I hope it will not be thought inconsistent with the declaration above made, of my wishing to avoid controversy, that I desire it may be understood that I consider the case just mentioned, as not affording the least proof of the Cow-pock being infectious by effluvia; no instance of such a case having fallen under my observation. I deemed this explanation necessary, although the object of my letter is to contradict the report of the fatal cases by the Cow-pock Inoculation.

